# Functions of Stress-Induced Lipid Droplets in the Nervous System

**DOI:** 10.3389/fcell.2022.863907

**Published:** 2022-04-14

**Authors:** Eva Islimye, Victor Girard, Alex P. Gould

**Affiliations:** Laboratory of Physiology and Metabolism, The Francis Crick Institute, London, United Kingdom

**Keywords:** lipid droplets, glia, neurons, neural stem cells, lipotoxicity, neurological disorders, cholesteryl esters and triacylglycerols

## Abstract

Lipid droplets are highly dynamic intracellular organelles that store neutral lipids such as cholesteryl esters and triacylglycerols. They have recently emerged as key stress response components in many different cell types. Lipid droplets in the nervous system are mostly observed *in vivo* in glia, ependymal cells and microglia. They tend to become more numerous in these cell types and can also form in neurons as a consequence of ageing or stresses involving redox imbalance and lipotoxicity. Abundant lipid droplets are also a characteristic feature of several neurodegenerative diseases. In this minireview, we take a cell-type perspective on recent advances in our understanding of lipid droplet metabolism in glia, neurons and neural stem cells during health and disease. We highlight that a given lipid droplet subfunction, such as triacylglycerol lipolysis, can be physiologically beneficial or harmful to the functions of the nervous system depending upon cellular context. The mechanistic understanding of context-dependent lipid droplet functions in the nervous system is progressing apace, aided by new technologies for probing the lipid droplet proteome and lipidome with single-cell type precision.

## Introduction

Lipid droplets (LDs) are intracellular organelles with a core of neutral lipids, such as triacylglycerols (TAGs) and cholesteryl esters (CEs), surrounded by a monolayer of charged phospholipids and proteins [reviewed in detail in (Walther and Farese, 2012; Wilfling et al., 2014; Walther et al., 2017; Olzmann and Carvalho, 2019)]. In brief, LDs bud from the endoplasmic reticulum (ER) and exchange lipids *via* direct contacts with several intracellular organelles including the mitochondria, ER, nucleus, peroxisomes and lysosomes [reviewed in detail in (Goodman, 2008; Gao and Goodman, 2015; Barbosa and Siniossoglou, 2017; Schuldiner and Bohnert, 2017; Geltinger et al., 2020; Herker et al., 2021; Thiam and Ikonen, 2021; Rakotonirina-Ricquebourg et al., 2022)]. LDs play a well-known role in adipocyte energy storage but are also implicated in a diverse range of other processes (Welte and Gould, 2017; Beller et al., 2020). For example, LDs can be induced in a wide range of different cell types in response to metabolic stresses such as excess dietary fat, starvation, hypoxia, and redox imbalance (Welte and Gould, 2017; Henne et al., 2018; de la Rosa Rodriguez and Kersten, 2020; Geltinger et al., 2020). LD accumulation in non-adipocyte cells is a hallmark of pathologies where there is lipotoxicity, including non-alcoholic fatty liver disease, obesity-related and diabetic kidney disease, as well as several cancers (Scorletti and Carr, 2022; Gluchowski et al., 2017; Opazo-Ríos et al., 2020; D'Agati et al., 2016; Krahmer et al., 2013; Petan, 2020; Nagarajan et al., 2021). In light of this, drugs targeting the synthesis of TAG, a major LD cargo, or other aspects of lipid metabolism are thought to provide useful therapeutic strategies for several diseases (Yoon et al., 2021).

**FIGURE 1:**
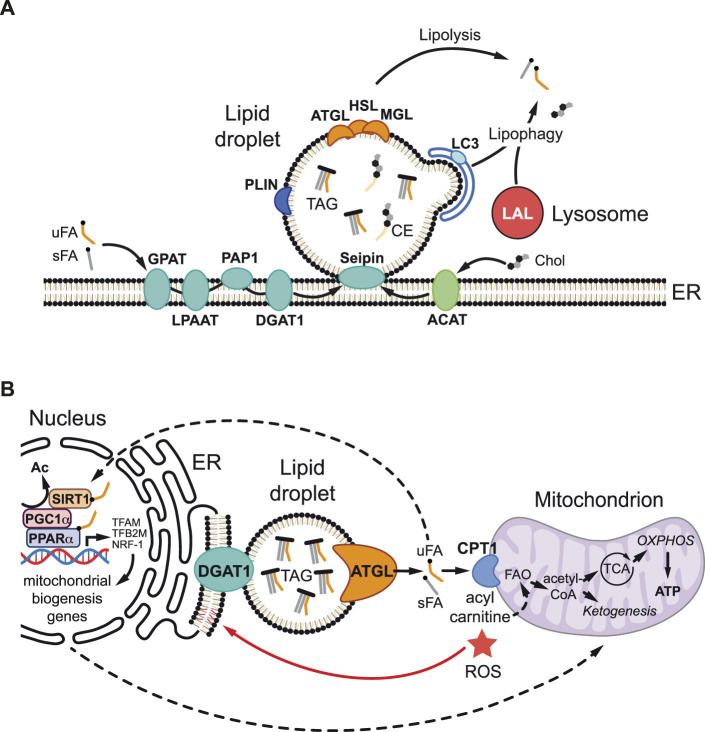
Lipid droplet metabolism and mitochondrial regulation. **(A)** During lipid droplet (LD) biogenesis, triacylglycerols (TAG) and cholesterol esters (CEs) are synthesized in the endoplasmic reticulum (ER). TAGs are generated from unsaturated and saturated fatty acids (uFAs and sFAs respectively) and glycerol-3-phosphate *via* four sequential enzymatic reactions involving glycerol-3-phosphate acyltransferase (GPAT), lysophosphatidic acid acyltransferase (LPAAT), phosphatidate phosphatase 1 (PAP1), and diacylglycerol acyltransferase 1 (DGAT1). CEs are generated by acyl-CoA cholesterol acyltransferase (ACAT), which esterifies FAs to cholesterol (Chol). ER-resident enzyme Seipin controls the channelling of newly synthesized neutral lipids into the growing LD core. TAG and CE accumulate between the two membrane leaflets of the ER bilayer, forming a nascent lipid lens that buds off as a LD. The LD surface is a phospholipid monolayer coated with a specific set of proteins including perilipins (PLIN), which maintain structure and regulate lipolysis, as well as adipocyte triglyceride lipase (ATGL), hormone sensitive lipase (HSL) and monoacylglycerol lipase (MAGL), which sequentially hydrolyze TAG to liberate free FAs *via* neutral lipolysis. During lipophagy, lysosomal acid lipases (LALs) hydrolyze TAG in the lysosome *via* acid lipolysis after phagophore engulfment involving microtubule-associated protein light chain 3 (LC3). **(B)** LDs can protect against lipotoxicity and high reactive oxygen species (ROS) *via* multiple non-mutually exclusive mechanisms. LDs buffer cytoplasmic free FA levels and generate lipid ligands/signals that stimulate the nuclear receptor peroxisome proliferator-activated receptor α (PPARα), a partner of PPARγ-Coactivator-1α (PGC1α), either *via* direct binding or indirectly *via* interaction with the sirtuin 1 (SIRT1) deacetylase. SIRT1 deacetylase removes an acetyl group (Ac) and activates PGC1α allowing it to partner with PPARα to promote the transcription of target genes involved in mitochondrial biogenesis and function, including Transcription factor A mitochondrial (TFAM), Transcription factor B2 mitochondrial (TFB2M), and Nuclear respiratory factor 1 (NRF-1). LDs also efficiently deliver FAs to mitochondria, where carnitine palmitoyltransferase (CPT1), converts them into acylcarnitines for fatty acid oxidation (FAO) to produce adenosine triphosphate (ATP), *via* the tricarboxylic acid (TCA) cycle and oxidative phosphorylation (OXPHOS), and also ketone bodies (ketogenesis). In addition, the environment of the LD core may minimize the potentially toxic effects of oxidized polyunsaturated FAs by protecting against lipid peroxidation or by sequestering already peroxidated lipids.

During LD biogenesis (Figure 1A), the synthesis of TAGs and CEs takes place within the phospholipid bilayer of the ER [reviewed in detail in (Walther et al., 2017; Olzmann and Carvalho, 2019; Heier and Kühnlein, 2018)]. TAGs are produced from fatty acids by four successive enzyme reactions that result in the esterification of three fatty acids to a glycerol backbone. The final step of TAG synthesis is catalyzed by diacylglycerol acyl transferases (DGAT1 and DGAT2). CEs are synthesized by esterification of fatty acids with cholesterol, a reaction catalyzed by acyl-CoA cholesterol acyltransferase (ACAT). TAGs and CEs tend to concentrate away from charged phospholipids, forming a neutral lipid lens between the ER membrane leaflets. Under the control of multiple ER-resident proteins such as Seipin, which forms oligomeric rings in the ER, neutral lipids are channelled into the growing LD core (Thiam and Ikonen, 2021). When new LDs form and bud off from the ER they become coated with a unique set of proteins, the LD proteome. This includes members of the Perilipin family that function to maintain LD integrity and to regulate LD lipolysis (Kimmel and Sztalryd, 2016). The catabolism of neutral lipids stored in the LD core is achieved by two major mechanisms, lipolysis and lipophagy (Figure 1A). During lipolysis, TAG lipases localized at the LD surface, such as Adipose Triglyceride Lipase (ATGL), liberate free fatty acids and diacylglycerol (Grabner et al., 2021). Diacylglycerol can be further hydrolyzed to produce additional free fatty acids by the sequential action of Hormone Sensitive Lipase (HSL) and Monoacylglycerol Lipase (MAGL). During lipophagy, neutral lipids are degraded by a selective form of autophagy in which LDs are engulfed by autophagosomes, which then fuse with acidic lysosomes so that TAGs and CEs can then be degraded by the lysosomal acidic lipases (Singh et al., 2009; Martinez-Lopez and Singh, 2015; Haidar et al., 2021).

In the mammalian brain, lipid metabolism is known to be highly cell-type specific (Fitzner et al., 2020). LDs have been reported in non-pathological *in vivo* contexts to localize mostly to ependymal cells (ependymocytes) and microglia (Lucken-Ardjomande Häsler et al., 2014; Hamilton et al., 2015; Shimabukuro et al., 2016; Hofmann et al., 2017; Fitzner et al., 2020; Marschallinger et al., 2020; Chausse et al., 2021; Loving et al., 2021; Ramosaj et al., 2021; Xu et al., 2021). LDs can also form in astrocytes, oligodendrocytes and pericytes of the neurovasculature but are predominantly observed *in vivo* in these mammalian glial subtypes during stress or pathological conditions (Shimabukuro et al., 2016; Farmer et al., 2020; Lee et al., 2021; Ralhan et al., 2021).

### Stresses and Diseases That Induce Lipid Droplets in the Nervous System

From as far back as Alois Alzheimer’s 1907 description of glial “adipose saccules”, numerous correlations have been made between LD accumulation in the brain and neurodegenerative diseases such as amyotrophic lateral sclerosis (ALS), Huntington’s disease, Parkinson’s disease and Alzheimer’s disease. The links between lipid metabolism, LDs and these neurodegenerative diseases have been discussed in detail in a number of recent reviews (Hamilton and Fernandes, 2018; Pennetta and Welte, 2018; Farmer et al., 2020; Haidar et al., 2021; Tadepalle and Rugarli, 2021; Teixeira et al., 2021). For some hereditary neurodegenerative conditions, causal links have been made to mutations in genes encoding proteins regulating LD biogenesis or turnover. For example, in the case of ALS caused by mutations in human Vesicle-Associated Membrane Protein (VAMP)-associated protein B (hVapB), combined human and *Drosophila* analyses implicate defective LD biogenesis as a contributory factor (Sanhueza et al., 2015; Pennetta and Welte, 2018; Farmer et al., 2020). Dominant mutations in an ER protein that regulates LD assembly, Seipin, can lead to seipinopathies including some forms of motor neuron disease (Windpassinger et al., 2004; Ito and Suzuki, 2009; Guo et al., 2013; Tadepalle and Rugarli, 2021). Related to this, the ER shaping factor Receptor Expression-Enhancing Protein 1 (REEP1) is required for forming appropriate numbers of LDs in the mouse brain and dominant mutations in this protein are associated with human hereditary spastic paraplegia (Züchner et al., 2006; Renvoisé et al., 2016). Furthermore, loss-of-function mutations in Sorting nexin 14 (Snx14), an ER-LD tethering protein, are associated with a form of spinocerebellar ataxia called SCAR20 (Datta et al., 2019; Datta et al., 2020). In the case of LD lipolysis, recessive mutations in a brain TAG lipase (DDHD Domain-Containing 2 (DDHD2)), which hydrolyzes LD core lipids, underlie a form of complex hereditary spastic paraplegia (Schuurs-Hoeijmakers et al., 2012; Inloes et al., 2018). Additionally, mutations in Huntingtin (Htt), a scaffold protein connecting the selective autophagy receptor p62 to LD cargo disrupt LD macroautophagy (lipophagy) and lead to Huntington’s disease (Rui et al., 2015). For many other neurodegenerative diseases, it is clear that lipid metabolism is perturbed but direct links between specific LD components and pathologies have not yet been made.

Many different stresses are known to induce LDs in the mammalian nervous system. In Schwann cells of the peripheral nervous system (PNS), infection by *Mycobacterium leprae* leads to myelin breakdown and Peroxisome Proliferator-Activated Receptor gamma (PPARγ)-dependent induction of LDs (Mattos et al., 2011; Díaz Acosta et al., 2018; Mietto et al., 2020). In the adult central nervous system (CNS), microglia accumulate LDs in response to innate inflammation, dietary high fat or low glucose, neurodegeneration, neuronal excitotoxicity or injury (Tamosaityte et al., 2016; Churchward et al., 2018; Chali et al., 2019; Ogrodnik et al., 2019; Raas et al., 2019; Marschallinger et al., 2020; Claes et al., 2021; Gouna et al., 2021; Zhuang et al., 2022). Microglia also accumulate LDs during ageing in the mouse and human brain, and this is associated with defective phagocytosis and a proinflammatory cell state (Marschallinger et al., 2020). Glial-like ependymal cells of the vertebrate CNS can also accumulate LDs in response to injury or a high fat diet (Enos et al., 2019; Maya-Monteiro et al., 2021). Moreover, astrocytes display increased LDs *in vivo* in response to a high fat diet and *ex vivo/in vitro* upon many different stresses including nutrient deprivation, hypoxia, excess fatty acids, γ-secretase inhibition, adrenergic receptor stimulation and neuronal excitotoxicity (Kwon et al., 2017; Ioannou et al., 2019; Ogrodnik et al., 2019; Gutierrez et al., 2020; Smolič et al., 2021). In the case of a high-fat diet, astrocytes of the hypothalamus that accumulate LDs also express proinflammatory cytokines and may therefore contribute to obesity-induced hypothalamic inflammation (Kwon et al., 2017).

In the invertebrate genetic model organism *Drosophila,* stress-induced LDs have been well characterized in both the developing and adult nervous systems (Bailey et al., 2015; Liu et al., 2015). In the developing CNS, LDs form predominantly in cortex and subperineurial glia, which constitute the niche for multipotent self-renewing neural stem cells called neuroblasts, and they increase following exposure to hypoxia or oxidant chemicals (Bailey et al., 2015; Kis et al., 2015; Dong et al., 2021). At adult stages, LDs are also present in glia of the CNS and increase during hypoxia (Smolič et al., 2021). In the adult retina, part of the PNS, several different genetic models of neurodegeneration lead to an increase in LDs in glial-like retinal pigment cells (RPCs) (Liu et al., 2015; Liu et al., 2017; Cabirol-Pol et al., 2018; Van Den Brink et al., 2018; Yeshaw et al., 2019; Girard et al., 2020; Muliyil et al., 2020). Sparse LDs have also been reported in *Drosophila* CNS and photoreceptor neurons and, in the latter, it is known that they increase in abundance in several neurodegeneration models (Van Den Brink et al., 2018; Wat et al., 2020; Girard et al., 2021). In both the mammalian and *Drosophil*a nervous systems, a growing body of evidence indicates that a common feature of many of the stresses and pathologies that induce glial LDs is redox imbalance, which is associated with high levels of reactive oxygen species (ROS) (Bailey et al., 2015; Liu et al., 2015; Liu et al., 2017; Ioannou et al., 2019; Cheng et al., 2020; Muliyil et al., 2020).

### Roles of Lipid Droplets in Glia

LDs perform a myriad of context-dependent cellular functions beyond energy homeostasis, including the storage of vitamin and signalling lipid precursors, the suppression of ER stress and lipotoxicity, as well as the maturation, storage, turnover and quality control of proteins [reviewed in (Welte and Gould, 2017; Roberts and Olzmann, 2020)]. In glia, the accumulation of abundant LDs tends to correlate with the presence of stress and disease (Section 2.1). In principle, therefore, LDs could either be a driver or a mitigator (albeit not a 100% efficient one) of cellular dysfunction. Consistent with this, beneficial and harmful roles have been ascribed to stress-induced glial LDs, depending upon biological context. In addition to contextual differences, it is challenging to assign specific functions to LDs as few, if any, genetic or pharmacological manipulations are completely specific for this organelle and interpreting phenotypes is not always straightforward. Nevertheless, some of the more specific perturbations of glial LDs have targeted the enzymes catalyzing the biosynthesis and lipolysis of their neutral lipid cargos—CEs and TAGs.

In the developing mammalian brain, cholesterol is abundant and the majority of it is synthesized in oligodendrocytes and utilized in myelination (Dietschy, 2009). In the adult brain, however, most cholesterol is synthesized in astrocytes and it can be transferred to neurons in order to maintain axonal integrity (Dietschy, 2009; Mou et al., 2020; Staurenghi et al., 2021). In mouse models of Alzheimer’s disease, brain CEs—as well as TAGs—are elevated and LDs accumulate in forebrain ependymal cells of the neural stem cell niche (Chan et al., 2012; Yang et al., 2014; Hamilton et al., 2015). LDs in microglia are also implicated as a recent study showed that a genetic risk factor for Alzheimer’s, the apolipoprotein E4 (ApoE4) allele, increases their abundance and also alters microglial properties such as phagocytosis (Machlovi et al., 2022). In several Alzheimer’s models, the enzyme synthesizing the CE cargo of LDs—ACAT also known as sterol O-acyltransferase (SOAT)—has been blocked using genetic or pharmacological methods. In the context of human mutant amyloid precursor protein (APP) and triple-transgenic mouse models, ACAT inhibition is beneficial as it substantially reduces APP processing and the production of extracellular amyloid plaques (Hutter-Paier et al., 2004; Shibuya et al., 2014). Brain CEs can also be lowered indirectly by converting cholesterol to 24 (S)-hydroxycholesterol, which can then be secreted from cells and eliminated *via* the blood brain barrier (Moutinho et al., 2016). Consistent with this, a chemical activator of cholesterol 24-hydroxylase (CYP46A1) increased 24-hydroxycholesterol secretion from APP mutant iPSC-derived neurons (but not astrocytes) lowering CEs and increasing proteosomal degradation of phosphorylated Tau, a hallmark of Alzheimer’s Disease (van der Kant et al., 2019). Although it is not yet clear how lowering CEs decreases APP processing and phospho-Tau degradation, altered trafficking and autophagy in microglia and neurons rather than in ependymal cells are likely to be relevant (Puglielli et al., 2001; Shibuya et al., 2014; Shibuya et al., 2015; van der Kant et al., 2019). In summary, the ACAT and CYP46A1 manipulations suggest that biosynthesis of the CE cargo of LDs can be harmful, contributing to the pathogenesis of Alzheimer’s disease.

Several recent *Drosophila* and mammalian studies have blocked glial LD accumulation by targeting TAG metabolism, using DGAT1 inhibition or ATGL overexpression (Bailey et al., 2015; Liu et al., 2015; Van Den Brink et al., 2018; Nakajima et al., 2019; Muliyil et al., 2020; Smolič et al., 2021). Comparisons between these studies provide some useful insights into the roles of glial LDs. The two *Drosophila* studies using DGAT1 (Mdy) knockdown both reported that this method of blocking glial LDs leads to non-cell autonomous cellular dysfunction: in one context late-onset adult photoreceptor degeneration and, in the other, underproliferation of neural stem cells and increased ROS during hypoxia (Bailey et al., 2015; Van Den Brink et al., 2018). Similarly, in cultured mammalian astrocytes, DGAT1 and/or DGAT2 inhibitors were used to block LDs. This resulted in a concomitant decrease in astrocyte cell number, suggesting that LD biosynthesis is important for glial proliferation and/or cell survival (Nakajima et al., 2019; Smolič et al., 2021). The outcomes of all four *Drosophila* and mammalian studies that inhibited DGAT1/2 are therefore consistent in showing that glial biosynthesis of TAGs can be beneficial in diverse contexts.

Three adult *Drosophila* retinal studies have utilized ATGL (Bmm) overexpression to boost TAG lipolysis and thus delete LDs. One found that this manipulation increases age-dependent photoreceptor degeneration (Van Den Brink et al., 2018). In contrast, the two other studies observed that ATGL overexpression substantially rescues photoreceptor degeneration in retinal cells mutant for a metalloprotease [A Disintegrin And Metalloproteinase Domain-Containing Protein 17 (ADAM17)/Tumor Necrosis Factor (TNF)-Alpha Converting Enzyme (TACE)] or for mitochondrial components [Mitofusin, Nicotinamide adenine dinucleotide (NADH) Dehydrogenase (ubiquinone) 42 kDa subunit (ND-42) or Methionyl-transfer Ribonucleic Acid (tRNA) synthetase, mitochondrial (MetRS-m)], leading the authors to conclude that, in these contexts, glial LDs promote neurodegeneration (Liu et al., 2015; Muliyil et al., 2020). However, a reinterpretation of this conclusion was recently suggested by systematic side-by-side comparisons of DGAT1 knockdown and ATGL overexpression, not in glia but in *Drosophila* renal cells (Lubojemska et al., 2021). This study showed that, although both manipulations efficiently block LD accumulation, the former is harmful whereas the latter is beneficial for cell function. Based on these and other findings, it was argued that overexpression of the lipid-droplet resident enzyme ATGL equates to a gain, not a loss, of an LD subfunction, enhancing the ability of the LD to stimulate TAG lipolysis (Lubojemska et al., 2021). This may also be the case in glia, such that DGAT1 and ATGL work in the same not opposite “directions” to promote a beneficial flux of fatty acids through the TAG compartment. More generally, the comparisons of DGAT1 and ATGL manipulations in different glial contexts illustrate that assigning an overall protective or a harmful role to LDs can be confusing and, at best, is an oversimplification. Instead, it may be useful to adopt a more nuanced approach, parsing the individual subfunctions of LDs using specific manipulations that avoid targeting more general aspects of lipid metabolism such as fatty acid synthesis or uptake.

A growing body of evidence is now shedding light on the mechanisms by which LDs in glia, and in other cell types, function to protect against lipotoxicity and redox imbalance (high ROS) during metabolic stress and disease (Ioannou et al., 2019; Bailey et al., 2015; Cheng et al., 2020; Lubojemska et al., 2021; Bensaad et al., 2014; Nguyen et al., 2017; Ackerman et al., 2018; Islam et al., 2019; Taïb et al., 2019; Liu et al., 2021). These protective LD roles appear to be intimately linked with mitochondria *via* at least four non-mutually exclusive mechanisms, whose relative importance is likely to be context dependent (Figure 1B). First, LDs in glia can provide an efficient conduit for delivering lipids *via* lipolysis or lipophagy to mitochondria for β-oxidation, in order to prevent fatty acid accumulation to toxic levels and/or to generate adenosine triphosphate (ATP) and ketone bodies (Rambold et al., 2015; Schulz et al., 2015; Ioannou et al., 2019; Wu et al., 2020). Second, this lipid trafficking route may help to minimize the potentially toxic effects of oxidized polyunsaturated fatty acids (PUFAs) *via* the LD core acting to protect against lipid peroxidation (Bailey et al., 2015; Li et al., 2018) or to sequester already peroxidated lipids (Liu et al., 2015; Ioannou et al., 2019). Third, in the context of mouse embryonic fibroblasts and glioma cells, LDs have been shown to act as a lipid buffer that is not required to deliver fatty acids to mitochondria but to sequester them, thus preventing acylcarnitine accumulation and lipotoxic dysregulation of mitochondria (Nguyen et al., 2017; Cheng et al., 2020). Fourth, LDs are also known, at least in non-neural contexts, to generate lipid signals that promote mitochondrial biogenesis and function (Haemmerle et al., 2011; Najt et al., 2020; Lubojemska et al., 2021). Hence, ATGL lipolysis at the surface of LDs can release fatty acids that activate the nuclear receptor PPARα, a partner of PPAR_γ_ Coactivator-1α (PGC1α), either directly or *via* the Sirtuin 1 deacetylase (Haemmerle et al., 2011; Najt et al., 2020; Lubojemska et al., 2021).

Distinct from a role in mitochondrial regulation, glial LDs can also regulate the activity of the intercellular signalling protein Hedgehog (Hh). In cortex glia of the developing *Drosophila* CNS, a proportion of the total Hh protein colocalizes with markers of the LD surface (Dong et al., 2021). Glial knockdown of a *Drosophila* Perilipin called Lipid storage droplet-2 (Lsd2), a negative regulator of ATGL, prevented glial overexpressed Hh from mediating an anti‐proliferative effect on neighbouring neural stem cells (Dong et al., 2021). It is therefore possible that the association of Hh with LDs modulates its secretion and/or activity.

### Intercellular Lipid Transfer and Glial Lipid Droplets

**FIGURE 2:**
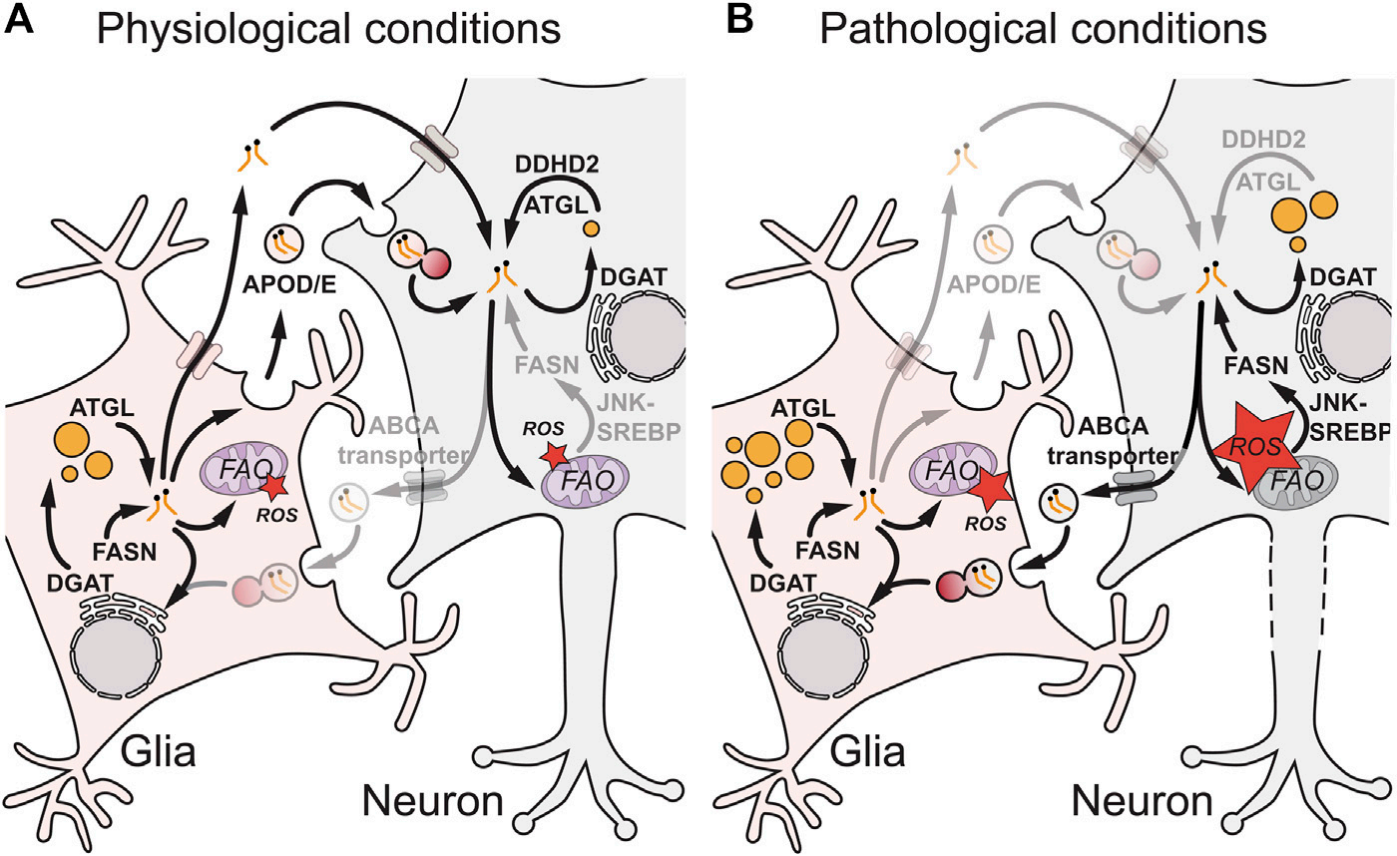
Glial-neuronal lipid transfer during physiological and pathological conditions. During physiological conditions **(A)**, the exchange of lipids between glia and neurons is mediated by apolipoprotein D/E (APOD/E) particles or other protein carriers such as albumin. In glia, fatty acids generated by fatty acid synthase (FASN) and converted into triacylglycerols (TAGs) *via* diacylglycerol acyltransferase (DGAT) can be remobilized from lipid droplets (LDs) by adipose triglyceride lipase (ATGL) for transfer to neurons or to enter mitochondria for fatty acid oxidation (FAO). In neurons, ATGL and DDHD Domain-Containing 2 (DDHD2) ensure that TAG lipolysis approximately matches TAG synthesis, preventing LD accumulation and ensuring the FA supply for neuronal functions such as membrane synthesis. Under pathological conditions **(B)**, mitochondrial dysfunction in neurons is associated with high reactive oxygen species (ROS) that trigger c-Jun N-terminal Kinase (JNK) and sterol regulatory element-binding protein (SREBP) signalling, which increases FASN synthesis of FAs and in some circumstances leads to ectopic neuronal LDs. Excess neuronal FAs are secreted from neurons *via* ATP-binding cassette (ABC) A transporters and APOD/E particles, taken up by glia *via* endocytosis and trafficked through the endolysosomal pathway and ER *via* DGAT into glial LDs. Glial LDs may protect against lipotoxicity and high reactive oxygen species (ROS) *via* multiple non-mutually exclusive mechanisms (Figure 1B). In neurons during pathological conditions, altered TAG metabolism and ectopic LDs may contribute to dysfunction and neurodegeneration (axonal dotted line).

Glia are known to secrete many types of lipids including cholesterol, fatty acids, phospholipids and phosphoglycolipids [reviewed in (Ralhan et al., 2021; Lee et al., 2021; Lane-Donovan et al., 2014; Mahley, 2016)]. These lipids are bound to extracellular proteins such as ApoE and can be taken up by neurons *via* low‐density lipoprotein (LDL) receptors or fatty acid transporters. They are known to be essential for the maintenance of multiple aspects of neuronal function, including membrane homeostasis, neurite outgrowth and intracellular signalling. Importantly, lipids can also be transferred in the reverse direction, from neurons to glia. A series of elegant papers (Ioannou et al., 2019; Liu et al., 2015; Liu et al., 2017; Van Den Brink et al., 2018; Muliyil et al., 2020; Moulton et al., 2021) used *Drosophila* and mammalian models of redox imbalance and neurodegeneration to demonstrate that metabolically stressed neurons deliver potentially toxic fatty acids to glia (Figure 2) [reviewed in detail in (Ralhan et al., 2021)]. In the context of *Drosophila* photoreceptor neurons, genetic knockdowns of mitochondrial components such as ND-42 generate redox imbalance, which activates c‐Jun‐N‐terminal Kinase (JNK) and Sterol Regulatory Element Binding Protein (SREBP) and stimulates the synthesis of fatty acids (Liu et al., 2015; Liu et al., 2017; Van Den Brink et al., 2018; Moulton et al., 2021). These fatty acids then become peroxidated in the presence of high ROS, exported from neurons *via* ATP-binding cassette transporter A (ABCA) transporters and transferred *via* an apolipoprotein D (ApoD) orthologue, Glaz, to glial-like RPCs. In RPCs, lipidated Glaz is thought to be taken up by an ApoD/E receptor, Lipoprotein Receptor-related Protein 1 (Lrp1), and fatty acids then trafficked in a clathrin-dependent manner *via* fatty acid transport protein (FatP) to be esterified *via* DGAT1 into TAGs stored in LDs (Liu et al., 2017; Van Den Brink et al., 2018; Moulton et al., 2021). Similarly, in a mammalian neuron-astrocyte coculture model, excitotoxicity was used to induce redox imbalance and increase autophagy, leading to neuronal production of excess free fatty acids (Ioannou et al., 2019). These fatty acids are then transferred to astrocytes *via* an ApoE and clathrin-dependent mechanism, where they are likely trafficked *via* the brain-specific fatty acid-binding protein, Fabp7, into LDs (Liu et al., 2017; Ioannou et al., 2019; Islam et al., 2019; Moulton et al., 2021; Qi et al., 2021). Together, the mammalian and *Drosophila* studies show that glia take up fatty acids form neurons and this can protect them from lipotoxicity. In this context, glial LDs can play a neuroprotective role, sequestering potentially toxic or peroxidated lipids (Liu et al., 2017; Ioannou et al., 2019; Moulton et al., 2021).

### Roles of Lipid Droplets in Neural Stem and Progenitor Cells

Neural stem and progenitor cells (NSPCs), like other stem cells, are regulated by many different aspects of lipid metabolism [reviewed in (Hamilton and Fernandes, 2018; Knobloch and Jessberger, 2017; Harkins et al., 2021; Madsen et al., 2021)]. Although NSPCs have been extensively characterized, there are very few reports of LDs in these cells *in vivo* in physiological wildtype conditions*.* However, in the embryonic mouse brain, conditional knockout in NSPCs of squalene synthase, an enzyme of cholesterol biosynthesis, results in LD accumulation and this correlates with the apoptosis of newborn neuronal progeny (Saito et al., 2009). These mutant embryonic NSPCs also upregulate vascular endothelial growth factor, although it is not clear if this process is linked to LDs (Saito et al., 2009). In the NSPC niches of the adult mammalian brain, LDs have mostly been described in niche cells such as ependymal cells not in the progenitors themselves [reviewed in (Ralhan et al., 2021; Hamilton and Fernandes, 2018)]. Recently, however, a study of the adult mouse brain found that NSPCs in the subventricular zone (SVZ) and dentate gyrus (DG) niches express the LD marker gene *perilipin 2* (*plin2*) and, when cultured *in vitro*, they accumulate abundant Plin2^+^ LDs (Ramosaj et al., 2021). In cultured SVZ NSPCs, Plin2^+^ LDs are smaller in size during the proliferative than the quiescent (non-dividing) state. Furthermore, Plin2^+^ LD content per NSPC varies and correlates positively with oxygen consumption and extracellular acidification rates as well as with proliferative ability (Ramosaj et al., 2021). NSPCs with more abundant LDs also tend to have higher ROS levels, although not an increase in lipid peroxidation. This suggests that LDs in NSPCs could safeguard PUFAs, as they are reported to do in glia of the developing *Drosophila* CNS (Bailey et al., 2015; Ramosaj et al., 2021). Interestingly, genetic or pharmacological knockdown of ATGL in cultured NSPCs increased LDs and led to a decrease in proliferation (Ramosaj et al., 2021). Conditional knockdown of fatty acid synthase (Fasn) in SVZ or DG NSPCs has the opposite effect on LDs, decreasing them, yet it also impairs proliferation (Knobloch et al., 2013; Ramosaj et al., 2021). Conversely, a Fasn gain-of-function mutation associated with a human cognitive disorder leads to an accumulation of TAGs and ER stress, again impairing the proliferation of DG NSPCs (Bowers et al., 2020). It is therefore tempting to speculate that fatty acid flux through the TAG compartment promotes NSPC proliferation. The beneficial role of TAG lipolysis in NSPCs could therefore be related to that observed in *Drosophila* glia (Section 2.3).

The effector mechanisms by which ATGL activity influences NSPC properties remain to be identified. One possibility is that TAG lipolysis provides an efficient route for delivering fatty acids to mitochondria for β-oxidation in order to fuel oxidative phosphorylation (Section 2.2; Figure 1B). For cultured SVZ NSPCs, however, the validity of this explanation is not yet clear, in part because there is no consensus on the contribution that fatty acid β-oxidation makes to overall oxygen consumption rate (Stoll et al., 2015; Ramosaj et al., 2021). Nevertheless, fatty acid import into mitochondria does play an important role in SVZ and DG NSPCs as strong pharmacological inhibition of a key enzyme in this pathway, carnitine palmitoyltransferase 1a (CPT1a), decreases their proliferation (Stoll et al., 2015; Knobloch et al., 2017). In addition, pharmacological and genetic approaches indicate that maintenance of the quiescent state of DG NSPCs *in vitro* and *in vivo* also requires CPT1a and, by inference mitochondrial β-oxidation (Knobloch et al., 2017). A connection between LDs and β-oxidation may also be important for NSPCs in the embryonic neocortex (Xie et al., 2016). In this context, LD lipolysis, carnitine biosynthesis and CPT1a are all required to maintain the pool size of Paired Box 6 (Pax6)^+^, T-box transcription factor Eomes/Tbr2^+^ neural stem cells. Carnitine biosynthesis and CPT1a were also shown to function in the balance between self-renewing and differentiative divisions and to maintain the mitochondrial redox balance of embryonic neural stem cells (Xie et al., 2016). Together, the available data suggest that, under physiological conditions, fatty acid flux through the LD compartment of NSPCs acts to promote mitochondrial β-oxidation, in turn regulating multiple stem cell properties including the cell division mode and proliferative state.

### Roles of Lipid Droplets in Neurons

Neurons in non-pathological and unstressed conditions tend to contain few if any LDs *in vivo*. An important question is why this is the case, given that neurons (and most other cell types) can form LDs *in vitro* when cultured under appropriate conditions. At least part of the explanation lies in the greater propensity of glia, ependymal cells and microglia to take up and process extracellular brain lipids (Sections 2.2 and 2.3). This *in vivo* “lipid sink” role has been mimicked in transwell co-cultures, where ectopic LDs in hippocampal neurons from ApoE3 or ApoE4 humanized mouse models of Alzheimer’s disease are cleared by astrocytes *via* ApoE-dependent extracellular lipid transport (Qi et al., 2021). Cell-intrinsic metabolic processes also make an important contribution towards preventing LDs from accumulating in neurons. For example, neurons express at least two different TAG lipases—ATGL and DDHD2—and their loss-of-function or chemical inhibition can lead to ectopic LDs in mammalian, *Drosophila* and *C. elegans* neurons (Inloes et al., 2014; Inloes et al., 2018; Yang et al., 2020a; Wat et al., 2020). In *C. elegans*, it has also been shown that mutations in an Abhydrolase Domain-Containing Protein 5 (ABHD5)/Comparative Gene Identification-58 (CGI-58) orthologue, a known co-activator of ATGL, or overexpression of DGAT1/2 orthologues leads to ectopic LDs in neurons (Yang et al., 2020a). Similarly, some perilipins protect LDs from lipolysis, and overexpression of either the Lsd-1 or Lsd-2 perilipins in *Drosophila* photoreceptors results in a large increase in the usually sparse LDs in these neurons (Girard et al., 2021). Collectively, these findings provide evidence that neurons do not usually accumulate LDs *in vivo,* because they actively turnover TAGs, favouring lipolysis over biosynthesis Figure 2. This raises the important general question of how neurons and other cell types regulate their rates of neutral lipid synthesis and lipolysis. In the case of TAGs in hepatocytes, an ER and LD-associated protein called hypoxia inducible lipid droplet associated (HILPDA) may contribute towards coordinating synthesis and lipolysis rates as it both stimulates DGAT1 and inhibits ATGL (de la Rosa Rodriguez et al., 2021). It is not yet clear whether or not HILPDA functions in similar way in neurons but it is known to be expressed and strongly hypoxia-inducible in human primary astrocytes (Allen et al., 2020). In neurons, neutral lipid turnover is likely to be beneficial for their function, at least during unstressed homeostatic conditions (Inloes et al., 2014; Inloes et al., 2018).

Neurons accumulate ectopic LDs in several neurodegenerative diseases and during ageing (Shimabukuro et al., 2016; Farmer et al., 2020; Conte et al., 2021). In sporadic and familial forms of Parkinson’s disease, α-synuclein in neurons aggregates into inclusion bodies known as Lewy bodies [reviewed in (Stok and Ashkenazi, 2020)]. It is linked to multiple aspects of lipid metabolism in complex ways, associating with LDs and directly binding phospholipids and unsaturated fatty acids [reviewed in (Teixeira et al., 2021; Roberts and Olzmann, 2020)]. The toxicity of α-synuclein in human induced pluripotent stem cell (iPSC) neurons likely involves unsaturated fatty acids as it is ameliorated by inhibitors of stearoyl-CoA desaturase (Vincent et al., 2018; Fanning et al., 2019). In the yeast *S. cerevisiae*, α-synuclein inhibits growth and this is rescued by inhibition of Pah1, a Lipin phosphatidate phosphatase, suggesting that diacylglycerol synthesis is harmful, contributing to toxicity (Soste et al., 2019). A closer functional link to TAG metabolism is suggested by a *Drosophila* model of Parkinson’s disease where human α-synuclein is expressed in photoreceptor neurons (Girard et al., 2021). Coexpression of the perilipin Lsd2 induces LDs in photoreceptors, which recruit α-synuclein to their surface and increases the proportion of protease-resistant α-synuclein, a characteristic associated with α-synuclein aggregation and neurodegeneration (Cremades et al., 2012; Suzuki et al., 2015; Girard et al., 2021). In a related α-synuclein photoreceptor model, co-expression of ATGL with α-synuclein decreased the protease-resistant fraction (Girard et al., 2021). Together, these findings suggest that TAG lipolysis in *Drosophila* photoreceptor neurons inhibits the formation of a toxic form of α-synuclein, thus playing a protective role. TAG lipolysis is also beneficial during the recovery of PNS neurons from optic nerve injury in mice, although it remains unclear if LDs accumulate (Yang et al., 2020b). Nevertheless, in this context, neuronal regeneration requires ATGL and DDHD2 but is inhibited by DGAT1/2 (Yang et al., 2020b). Given that phospholipid synthesis enzymes can also facilitate regeneration, it may be that redirecting fatty acids away from TAGs into membrane lipids is beneficial for neuronal regrowth (Yang et al., 2020b; Roy and Tedeschi, 2021). TAG lipolysis in neurons is not, however, universally beneficial. In a *C. elegans* genetic model of excitotoxicity, LDs accumulate in neurons during their degeneration but this is rescued by inactivation of ATGL or ABHD5/CGI-58 and worsened by C20 PUFA incorporation into phospholipids (Yang et al., 2020a). Hence, in this excitotoxic model, TAG lipolysis in neurons exacerbates their degeneration, perhaps because it redirects PUFA into membranes where they are vulnerable to peroxidation. ATGL-dependent lipolysis also appears to be detrimental in cultured mammalian motor neurons expressing Seipin N88S, a mutant associated with a dominant spastic paraplegia that localizes to LDs and induces ER stress (Holtta-Vuori et al., 2013). In summary, there appears to be no universal truth about whether TAG lipolysis in neurons protects or harms from stress or disease—it all depends upon biological context.

### Conclusion and Outlook

LDs are a common feature of the developing and adult nervous systems of vertebrates and invertebrates. They have been observed in essentially all major cell types of the nervous system, albeit to differing degrees and in some cases only in stress or disease contexts. Under physiological conditions *in vivo*, LDs primarily accumulate in glia, ependymal cells and microglia. However, even in the absence of detectable LDs, cells such as neurons are still actively turning over neutral lipids. LDs in the nervous system become more numerous as a hallmark of several neurodegenerative diseases and also as a consequence of stresses involving redox imbalance and lipotoxicity. LDs in the nervous system during health and disease participate in multiple complex functions, which are dependent upon the cell type that they accumulate in. It is often difficult to make conclusions about whether LDs overall are beneficial or harmful. A more useful approach is to assign functions to individual biochemical reactions that are directly linked to LDs, such as TAG synthesis or lipolysis.

Looking forwards, a central challenge is to understand how lipid metabolic networks become wired differently in glia, neural stem cells and neurons, and how this influences adaptation to stress and disease. An important part of addressing this issue will be to determine how stresses change the LD proteome differently in each cell type of the nervous system. Rapid advances in technologies such as spatially-resolved and single-cell transcriptomics and proteomics are likely to help greatly in this quest (Armand et al., 2021; Goto-Silva and Junqueira, 2021; Maniatis et al., 2021). In addition, comparisons between the LD proteomes of glia, ependymal cells, microglia and neurons may also be facilitated by genetically encoded strategies that provide cell-type specific proximity labelling using Ascorbate Peroxidase 2 (APEX2) or related enzymes (Bersuker et al., 2018). A surface analysis technology from the physical sciences, mass spectrometry imaging, is an approach that promises to offer lipidomics and perhaps even proteomics with single-cell resolution in brain tissue sections (Gilmore et al., 2019; Taylor et al., 2021). Several different mass spectrometry imaging (MSI) platforms have already been used to spatially resolve amino acid, protein, lipid and LD metabolism, where they are beginning to deliver exciting new insights into regional and cell-type specific features of brain metabolism (Steinhauser et al., 2012; Bailey et al., 2015; Narendra et al., 2020; Newell et al., 2020; Wang et al., 2022).

## References

[B1] Ackerman D. Tumanov S. Qiu B. Michalopoulou E. Spata M. Azzam A. (2018). Triglycerides Promote Lipid Homeostasis during Hypoxic Stress by Balancing Fatty Acid Saturation. Cell Rep. 24 (10), 2596–2605. e5. 10.1016/j.celrep.2018.08.015 30184495 PMC6137821

[B2] Allen S. P. Seehra R. S. Heath P. R. Hall B. P. C. Bates J. Garwood C. J. (2020). Transcriptomic Analysis of Human Astrocytes *In Vitro* Reveals Hypoxia-Induced Mitochondrial Dysfunction, Modulation of Metabolism, and Dysregulation of the Immune Response. Ijms 21 (21), 8028. 10.3390/ijms21218028 PMC7672558 33126586

[B3] Armand E. J. Li J. Xie F. Luo C. Mukamel E. A. (2021). Single-Cell Sequencing of Brain Cell Transcriptomes and Epigenomes. Neuron 109 (1), 11–26. 10.1016/j.neuron.2020.12.010 33412093 PMC7808568

[B4] Bailey A. P. Koster G. Guillermier C. Hirst E. M. A. MacRae J. I. Lechene C. P. (2015). Antioxidant Role for Lipid Droplets in a Stem Cell Niche of Drosophila. Cell 163 (2), 340–353. 10.1016/j.cell.2015.09.020 26451484 PMC4601084

[B5] Barbosa A. D. Siniossoglou S. (2017). Function of Lipid Droplet-Organelle Interactions in Lipid Homeostasis. Biochim. Biophys. Acta (Bba) - Mol. Cell Res. 1864 (9), 1459–1468. 10.1016/j.bbamcr.2017.04.001 28390906

[B6] Beller M. Herker E. Füllekrug J. (2020). Grease On-Perspectives in Lipid Droplet Biology. Semin. Cell Developmental Biol. 108, 94–101. 10.1016/j.semcdb.2020.06.017 32636101

[B7] Bensaad K. Favaro E. Lewis C. A. Peck B. Lord S. Collins J. M. (2014). Fatty Acid Uptake and Lipid Storage Induced by HIF-1α Contribute to Cell Growth and Survival after Hypoxia-Reoxygenation. Cell Rep. 9 (1), 349–365. 10.1016/j.celrep.2014.08.056 25263561

[B8] Bersuker K. Peterson C. W. H. To M. Sahl S. J. Savikhin V. Grossman E. A. (2018). A Proximity Labeling Strategy Provides Insights into the Composition and Dynamics of Lipid Droplet Proteomes. Developmental Cell 44 (1), 97–112. 10.1016/j.devcel.2017.11.020 29275994 PMC5764092

[B9] Bowers M. Liang T. Gonzalez-Bohorquez D. Zocher S. Jaeger B. N. Kovacs W. J. (2020). FASN-dependent Lipid Metabolism Links Neurogenic Stem/Progenitor Cell Activity to Learning and Memory Deficits. Cell Stem Cell 27 (1), 98–109. e11. 10.1016/j.stem.2020.04.002 32386572

[B10] Cabirol-Pol M.-J. Khalil B. Rival T. Faivre-Sarrailh C. Besson M. T. (2018). Glial Lipid Droplets and Neurodegeneration in aDrosophilamodel of Complex I Deficiency. Glia 66 (4), 874–888. 10.1002/glia.23290 29285794

[B11] Chali F. Milior G. Marty S. Morin-Brureau M. Le Duigou C. Savary E. (2019). Lipid Markers and Related Transcripts during Excitotoxic Neurodegeneration in Kainate-Treated Mice. Eur. J. Neurosci. 50 (1), 1759–1778. 10.1111/ejn.14375 30767299

[B12] Chan R. B. Oliveira T. G. Cortes E. P. Honig L. S. Duff K. E. Small S. A. (2012). Comparative Lipidomic Analysis of Mouse and Human Brain with Alzheimer Disease. J. Biol. Chem. 287 (4), 2678–2688. 10.1074/jbc.m111.274142 22134919 PMC3268426

[B13] Chausse B. Kakimoto P. A. Kann O. (2021). Microglia and Lipids: How Metabolism Controls Brain Innate Immunity. Semin. Cell Developmental Biol. 112, 137–144. 10.1016/j.semcdb.2020.08.001 32807643

[B14] Cheng X. Geng F. Pan M. Wu X. Zhong Y. Wang C. (2020). Targeting DGAT1 Ameliorates Glioblastoma by Increasing Fat Catabolism and Oxidative Stress. Cell Metab. 32 (2), 229–242. 10.1016/j.cmet.2020.06.002 32559414 PMC7415721

[B15] Churchward M. A. Tchir D. R. Todd K. G. (2018). Microglial Function during Glucose Deprivation: Inflammatory and Neuropsychiatric Implications. Mol. Neurobiol. 55 (2), 1477–1487. 10.1007/s12035-017-0422-9 28176274 PMC5820372

[B16] Claes C. Danhash E. P. Hasselmann J. Chadarevian J. P. Shabestari S. K. England W. E. (2021). Plaque-associated Human Microglia Accumulate Lipid Droplets in a Chimeric Model of Alzheimer's Disease. Mol. Neurodegeneration 16 (1), 50. 10.1186/s13024-021-00473-0 PMC8305935 34301296

[B17] Conte M. Medici V. Malagoli D. Chiariello A. Cirrincione A. Davin A. (2021). Expression Pattern of Perilipins in Human Brain during Aging and in Alzheimer's Disease. Neuropathol. Appl. Neurobiol. 48, e12756. 10.1111/nan.12756 34312912 PMC9291275

[B18] Cremades N. Cohen S. I. A. Deas E. Abramov A. Y. Chen A. Y. Orte A. (2012). Direct Observation of the Interconversion of Normal and Toxic Forms of α-Synuclein. Cell 149 (5), 1048–1059. 10.1016/j.cell.2012.03.037 22632969 PMC3383996

[B19] D'Agati V. D. Chagnac A. de Vries A. P. J. Levi M. Porrini E. Herman-Edelstein M. (2016). Obesity-related Glomerulopathy: Clinical and Pathologic Characteristics and Pathogenesis. Nat. Rev. Nephrol. 12 (8), 453–471. 10.1038/nrneph.2016.75 27263398

[B20] Datta S. Bowerman J. Hariri H. Ugrankar R. Eckert K. M. Corley C. (2020). Snx14 Proximity Labeling Reveals a Role in Saturated Fatty Acid Metabolism and ER Homeostasis Defective in SCAR20 Disease. Proc. Natl. Acad. Sci. U.S.A. 117, 33282–33294. 10.1073/pnas.2011124117 PMC7777019 33310904

[B21] Datta S. Liu Y. Hariri H. Bowerman J. Henne W. M. (2019). Cerebellar Ataxia Disease-Associated Snx14 Promotes Lipid Droplet Growth at ER-Droplet Contacts. J. Cell Biol 218 (4), 1335–1351. 10.1083/jcb.201808133 30765438 PMC6446855

[B22] de la Rosa Rodriguez M. A. Deng L. Gemmink A. van Weeghel M. Aoun M. L. Warnecke C. (2021). Hypoxia-inducible Lipid Droplet-Associated Induces DGAT1 and Promotes Lipid Storage in Hepatocytes. Mol. Metab. 47, 101168. 10.1016/j.molmet.2021.101168 33465519 PMC7881268

[B23] de la Rosa Rodriguez M. A. Kersten S. (2020). Regulation of Lipid Droplet Homeostasis by Hypoxia Inducible Lipid Droplet Associated HILPDA. Biochim. Biophys. Acta (Bba) - Mol. Cell Biol. Lipids 1865 (9), 158738. 10.1016/j.bbalip.2020.158738 32417386

[B24] Díaz Acosta C. C. Dias A. A. Rosa T. L. S. A. Batista-Silva L. R. Rosa P. S. Toledo-Pinto T. G. (2018). PGL I Expression in Live Bacteria Allows Activation of a CD206/PPARγ Cross-Talk that May Contribute to Successful *Mycobacterium leprae* Colonization of Peripheral Nerves. Plos Pathog. 14 (7), e1007151. 10.1371/journal.ppat.1007151 29979790 PMC6056075

[B25] Dietschy J. M. (2009). Central Nervous System: Cholesterol Turnover, Brain Development and Neurodegeneration. Biol. Chem. 390 (4), 287–293. 10.1515/BC.2009.035 19166320 PMC3066069

[B26] Dong Q. Zavortink M. Froldi F. Golenkina S. Lam T. Cheng L. Y. (2021). Glial Hedgehog Signalling and Lipid Metabolism Regulate Neural Stem Cell Proliferation in Drosophila. EMBO Rep. 22 (5), e52130. 10.15252/embr.202052130 33751817 PMC8097363

[B27] Enos N. Takenaka H. Scott S. Salfity H. V. N. Kirk M. Egar M. W. (2019). Meningeal Foam Cells and Ependymal Cells in Axolotl Spinal Cord Regeneration. Front. Immunol. 10, 2558. 10.3389/fimmu.2019.02558 31736973 PMC6838144

[B28] Fanning S. Haque A. Imberdis T. Baru V. Barrasa M. I. Nuber S. (2019). Lipidomic Analysis of α-Synuclein Neurotoxicity Identifies Stearoyl CoA Desaturase as a Target for Parkinson Treatment. Mol. Cell 73 (5), 1001–1014. 10.1016/j.molcel.2018.11.028 30527540 PMC6408259

[B29] Farmer B. C. Walsh A. E. Kluemper J. C. Johnson L. A. (2020). Lipid Droplets in Neurodegenerative Disorders. Front. Neurosci. 14, 742. 10.3389/fnins.2020.00742 32848541 PMC7403481

[B30] Fitzner D. Bader J. M. Penkert H. Bergner C. G. Su M. Weil M.-T. (2020). Cell-Type- and Brain-Region-Resolved Mouse Brain Lipidome. Cell Rep. 32 (11), 108132. 10.1016/j.celrep.2020.108132 32937123

[B31] Gao Q. Goodman J. M. (2015). The Lipid Droplet-A Well-Connected Organelle. Front. Cell Dev. Biol. 3, 49. 10.3389/fcell.2015.00049 26322308 PMC4533013

[B32] Geltinger F. Schartel L. Wiederstein M. Tevini J. Aigner E. Felder T. K. (2020). Friend or Foe: Lipid Droplets as Organelles for Protein and Lipid Storage in Cellular Stress Response, Aging and Disease. Molecules 25 (21), 5053. 10.3390/molecules25215053 PMC7663626 33143278

[B33] Gilmore I. S. Heiles S. Pieterse C. L. (2019). Metabolic Imaging at the Single-Cell Scale: Recent Advances in Mass Spectrometry Imaging. Annu. Rev. Anal. Chem. 12 (1), 201–224. 10.1146/annurev-anchem-061318-115516 30848927

[B34] Girard V. Goubard V. Querenet M. Seugnet L. Pays L. Nataf S. (2020). Spen Modulates Lipid Droplet Content in Adult Drosophila Glial Cells and Protects against Paraquat Toxicity. Sci. Rep. 10 (1), 20023. 10.1038/s41598-020-76891-9 33208773 PMC7674452

[B35] Girard V. Jollivet F. Knittelfelder O. Celle M. Arsac J.-N. Chatelain G. (2021). Abnormal Accumulation of Lipid Droplets in Neurons Induces the Conversion of Alpha-Synuclein to Proteolytic Resistant Forms in a Drosophila Model of Parkinson's Disease. Plos Genet. 17 (11), e1009921. 10.1371/journal.pgen.1009921 34788284 PMC8635402

[B36] Gluchowski N. L. Becuwe M. Walther T. C. Farese R. V. Jr. (2017). Lipid Droplets and Liver Disease: from Basic Biology to Clinical Implications. Nat. Rev. Gastroenterol. Hepatol. 14 (6), 343–355. 10.1038/nrgastro.2017.32 28428634 PMC6319657

[B37] Goodman J. M. (2008). The Gregarious Lipid Droplet. J. Biol. Chem. 283 (42), 28005–28009. 10.1074/jbc.r800042200 18611863 PMC2568941

[B38] Goto-Silva L. Junqueira M. (2021). Single-cell Proteomics: A Treasure Trove in Neurobiology. Biochim. Biophys. Acta (Bba) - Proteins Proteomics 1869 (7), 140658. 10.1016/j.bbapap.2021.140658 33845200

[B39] Gouna G. Klose C. Bosch-Queralt M. Liu L. Gokce O. Schifferer M. (2021). TREM2-dependent Lipid Droplet Biogenesis in Phagocytes Is Required for Remyelination. J. Exp. Med. 218 (10). 10.1084/jem.20210227 PMC8404472 34424266

[B40] Grabner G. F. Xie H. Schweiger M. Zechner R. (2021). Lipolysis: Cellular Mechanisms for Lipid Mobilization from Fat Stores. Nat. Metab. 3 (11), 1445–1465. 10.1038/s42255-021-00493-6 34799702

[B41] Guo J. Qiu W. Soh S. L. Y. Wei S. Radda G. K. Ong W.-Y. (2013). Motor Neuron Degeneration in a Mouse Model of Seipinopathy. Cell Death Dis 4, e535. 10.1038/cddis.2013.64 23470542 PMC3613842

[B42] Gutierrez E. Lütjohann D. Kerksiek A. Fabiano M. Oikawa N. Kuerschner L. (2020). Importance of γ-secretase in the Regulation of Liver X Receptor and Cellular Lipid Metabolism. Life Sci. Alliance 3 (6). 10.26508/lsa.201900521 PMC7195048 32354700

[B43] Haemmerle G. Moustafa T. Woelkart G. Büttner S. Schmidt A. van de Weijer T. (2011). ATGL-mediated Fat Catabolism Regulates Cardiac Mitochondrial Function via PPAR-α and PGC-1. Nat. Med. 17 (9), 1076–1085. 10.1038/nm.2439 21857651 PMC3244833

[B44] Haidar M. Loix M. Bogie J. F. J. Hendriks J. J. A. (2021). Lipophagy: a New Player in CNS Disorders. Trends Endocrinol. Metab. 32 (11), 941–951. 10.1016/j.tem.2021.08.010 34561114

[B45] Hamilton L. K. Dufresne M. Joppé S. E. Petryszyn S. Aumont A. Calon F. (2015). Aberrant Lipid Metabolism in the Forebrain Niche Suppresses Adult Neural Stem Cell Proliferation in an Animal Model of Alzheimer's Disease. Cell Stem Cell 17 (4), 397–411. 10.1016/j.stem.2015.08.001 26321199

[B46] Hamilton L. K. Fernandes K. J. L. (2018). Neural Stem Cells and Adult Brain Fatty Acid Metabolism: Lessons from the 3xTg Model of Alzheimer's Disease. Biol. Cell 110 (1), 6–25. 10.1111/boc.201700037 28980327

[B47] Harkins D. Cooper H. M. Piper M. (2021). The Role of Lipids in Ependymal Development and the Modulation of Adult Neural Stem Cell Function during Aging and Disease. Semin. Cell Developmental Biol. 112, 61–68. 10.1016/j.semcdb.2020.07.018 32771376

[B48] Heier C. Kühnlein R. P. (2018). Triacylglycerol Metabolism in *Drosophila melanogaster* . Genetics 210 (4), 1163–1184. 10.1534/genetics.118.301583 30523167 PMC6283168

[B49] Henne W. M. Reese M. L. Goodman J. M. (2018). The Assembly of Lipid Droplets and Their Roles in Challenged Cells. Embo j 37 (12). 10.15252/embj.201898947 PMC6003646 29789390

[B50] Herker E. Vieyres G. Beller M. Krahmer N. Bohnert M. (2021). Lipid Droplet Contact Sites in Health and Disease. Trends Cell Biol. 31 (5), 345–358. 10.1016/j.tcb.2021.01.004 33546922

[B51] Hofmann K. Rodriguez-Rodriguez R. Gaebler A. Casals N. Scheller A. Kuerschner L. (2017). Astrocytes and Oligodendrocytes in Grey and white Matter Regions of the Brain Metabolize Fatty Acids. Sci. Rep. 7 (1), 10779. 10.1038/s41598-017-11103-5 28883484 PMC5589817

[B52] Holtta-Vuori M. Salo V. T. Ohsaki Y. Suster M. L. Ikonen E. (2013). Alleviation of Seipinopathy-Related ER Stress by Triglyceride Storage. Hum. Mol. Genet. 22 (6), 1157–1166. 10.1093/hmg/dds523 23250914

[B53] Hutter-Paier B. Huttunen H. J. Puglielli L. Eckman C. B. Kim D. Y. Hofmeister A. (2004). The ACAT Inhibitor CP-113,818 Markedly Reduces Amyloid Pathology in a Mouse Model of Alzheimer's Disease. Neuron 44 (2), 227–238. 10.1016/j.neuron.2004.08.043 15473963

[B54] Inloes J. M. Hsu K.-L. Dix M. M. Viader A. Masuda K. Takei T. (2014). The Hereditary Spastic Paraplegia-Related Enzyme DDHD2 Is a Principal Brain Triglyceride Lipase. Proc. Natl. Acad. Sci. U.S.A. 111 (41), 14924–14929. 10.1073/pnas.1413706111 25267624 PMC4205627

[B55] Inloes J. M. Kiosses W. B. Wang H. Walther T. C. Farese R. V. Jr. Cravatt B. F. (2018). Functional Contribution of the Spastic Paraplegia-Related Triglyceride Hydrolase DDHD2 to the Formation and Content of Lipid Droplets. Biochemistry 57 (5), 827–838. 10.1021/acs.biochem.7b01028 29278326 PMC5854151

[B56] Ioannou M. S. Jackson J. Sheu S.-H. Chang C.-L. Weigel A. V. Liu H. (2019). Neuron-Astrocyte Metabolic Coupling Protects against Activity-Induced Fatty Acid Toxicity. Cell 177 (6), 1522–1535. e14. 10.1016/j.cell.2019.04.001 31130380

[B57] Islam A. Kagawa Y. Miyazaki H. Shil S. K. Umaru B. A. Yasumoto Y. (2019). FABP7 Protects Astrocytes against ROS Toxicity via Lipid Droplet Formation. Mol. Neurobiol. 56 (8), 5763–5779. 10.1007/s12035-019-1489-2 30680690

[B58] Ito D. Suzuki N. (2009). Seipinopathy: a Novel Endoplasmic Reticulum Stress-Associated Disease. Brain 132 (Pt 1), 8–15. 10.1093/brain/awn216 18790819

[B59] Kimmel A. R. Sztalryd C. (2016). The Perilipins: Major Cytosolic Lipid Droplet-Associated Proteins and Their Roles in Cellular Lipid Storage, Mobilization, and Systemic Homeostasis. Annu. Rev. Nutr. 36, 471–509. 10.1146/annurev-nutr-071813-105410 27431369

[B60] Kis V. Barti B. Lippai M. Sass M. (2015). Specialized Cortex Glial Cells Accumulate Lipid Droplets in *Drosophila melanogaster* . PLoS One 10 (7), e0131250. 10.1371/journal.pone.0131250 26148013 PMC4493057

[B61] Knobloch M. Braun S. M. G. Zurkirchen L. von Schoultz C. Zamboni N. Araúzo-Bravo M. J. (2013). Metabolic Control of Adult Neural Stem Cell Activity by Fasn-dependent Lipogenesis. Nature 493 (7431), 226–230. 10.1038/nature11689 23201681 PMC3587167

[B62] Knobloch M. Jessberger S. (2017). Metabolism and Neurogenesis. Curr. Opin. Neurobiol. 42, 45–52. 10.1016/j.conb.2016.11.006 27915086

[B63] Knobloch M. Pilz G.-A. Ghesquière B. Kovacs W. J. Wegleiter T. Moore D. L. (2017). A Fatty Acid Oxidation-dependent Metabolic Shift Regulates Adult Neural Stem Cell Activity. Cell Rep. 20 (9), 2144–2155. 10.1016/j.celrep.2017.08.029 28854364 PMC5583518

[B64] Krahmer N. Farese R. V. Jr. Walther T. C. (2013). Balancing the Fat: Lipid Droplets and Human Disease. EMBO Mol. Med. 5 (7), 973–983. 10.1002/emmm.201100671 23740690 PMC3721468

[B65] Kwon Y.-H. Kim J. Kim C.-S. Tu T. H. Kim M.-S. Suk K. (2017). Hypothalamic Lipid-Laden Astrocytes Induce Microglia Migration and Activation. FEBS Lett. 591 (12), 1742–1751. 10.1002/1873-3468.12691 28542876

[B66] Lane-Donovan C. Philips G. T. Herz J. (2014). More Than Cholesterol Transporters: Lipoprotein Receptors in CNS Function and Neurodegeneration. Neuron 83 (4), 771–787. 10.1016/j.neuron.2014.08.005 25144875 PMC4240629

[B67] Lee J. A. Hall B. Allsop J. Alqarni R. Allen S. P. (2021). Lipid Metabolism in Astrocytic Structure and Function. Semin. Cell Developmental Biol. 112, 123–136. 10.1016/j.semcdb.2020.07.017 32773177

[B68] Li N. Sancak Y. Frasor J. Atilla-Gokcumen G. E. (2018). A Protective Role for Triacylglycerols during Apoptosis. Biochemistry 57 (1), 72–80. 10.1021/acs.biochem.7b00975 29188717 PMC5975242

[B69] Liu L. Zhang K. Sandoval H. Yamamoto S. Jaiswal M. Sanz E. (2015). Glial Lipid Droplets and ROS Induced by Mitochondrial Defects Promote Neurodegeneration. Cell 160 (1-2), 177–190. 10.1016/j.cell.2014.12.019 25594180 PMC4377295

[B70] Liu L. MacKenzie K. R. Putluri N. Maletić-Savatić M. Bellen H. J. (2017). The Glia-Neuron Lactate Shuttle and Elevated ROS Promote Lipid Synthesis in Neurons and Lipid Droplet Accumulation in Glia via APOE/D. Cell Metab. 26 (5), 719–737. e6. 10.1016/j.cmet.2017.08.024 28965825 PMC5677551

[B71] Liu R. Lee J.-H. Li J. Yu R. Tan L. Xia Y. (2021). Choline Kinase Alpha 2 Acts as a Protein Kinase to Promote Lipolysis of Lipid Droplets. Mol. Cell 81 (13), 2722–2735. 10.1016/j.molcel.2021.05.005 34077757

[B72] Loving B. A. Tang M. Neal M. C. Gorkhali S. Murphy R. Eckel R. H. (2021). Lipoprotein Lipase Regulates Microglial Lipid Droplet Accumulation. Cells 10 (2), 198. 10.3390/cells10020198 33498265 PMC7909280

[B73] Lubojemska A. Stefana M. I. Sorge S. Bailey A. P. Lampe L. Yoshimura A. (2021). Adipose Triglyceride Lipase Protects Renal Cell Endocytosis in a Drosophila Dietary Model of Chronic Kidney Disease. Plos Biol. 19 (5), e3001230. 10.1371/journal.pbio.3001230 33945525 PMC8121332

[B74] Lucken-Ardjomande Häsler S. Vallis Y. Jolin H. E. McKenzie A. N. McMahon H. T. (2014). GRAF1a Is a Brain-specific Protein that Promotes Lipid Droplet Clustering and Growth, and Is Enriched at Lipid Droplet Junctions. J. Cell Sci 127 (Pt 21), 4602–4619. 10.1242/jcs.147694 25189622 PMC4215711

[B75] Machlovi S. I. Neuner S. M. Hemmer B. M. Khan R. Liu Y. Huang M. (2022). APOE4 Confers Transcriptomic and Functional Alterations to Primary Mouse Microglia. Neurobiol. Dis. 164, 105615. 10.1016/j.nbd.2022.105615 35031484 PMC8934202

[B76] Madsen S. Ramosaj M. Knobloch M. (2021). Lipid Metabolism in Focus: How the Build-Up and Breakdown of Lipids Affects Stem Cells. Development 148 (10), dev191924. 10.1242/dev.191924 34042969

[B77] Mahley R. W. (2016). Central Nervous System Lipoproteins. Atvb 36 (7), 1305–1315. 10.1161/atvbaha.116.307023 PMC4942259 27174096

[B78] Maniatis S. Petrescu J. Phatnani H. (2021). Spatially Resolved Transcriptomics and its Applications in Cancer. Curr. Opin. Genet. Development 66, 70–77. 10.1016/j.gde.2020.12.002 PMC7969406 33434721

[B79] Marschallinger J. Iram T. Zardeneta M. Lee S. E. Lehallier B. Haney M. S. (2020). Lipid-droplet-accumulating Microglia Represent a Dysfunctional and Proinflammatory State in the Aging Brain. Nat. Neurosci. 23 (2), 194–208. 10.1038/s41593-019-0566-1 31959936 PMC7595134

[B80] Martinez-Lopez N. Singh R. (2015). Autophagy and Lipid Droplets in the Liver. Annu. Rev. Nutr. 35, 215–237. 10.1146/annurev-nutr-071813-105336 26076903 PMC7909712

[B81] Mattos K. A. Lara F. A. Oliveira V. G. C. Rodrigues L. S. D'Avila H. Melo R. C. N. (2011). Modulation of Lipid Droplets by *Mycobacterium leprae* in Schwann Cells: a Putative Mechanism for Host Lipid Acquisition and Bacterial Survival in Phagosomes. Cell Microbiol 13 (2), 259–273. 10.1111/j.1462-5822.2010.01533.x 20955239

[B82] Maya-Monteiro C. M. Corrêa-da-Silva F. Hofmann S. S. Hesselink M. K. C. la Fleur S. E. Yi C. X. (2021). Lipid Droplets Accumulate in the Hypothalamus of Mice and Humans with and without Metabolic Diseases. Neuroendocrinology 111 (3), 263–272. 10.1159/000508735 32422642

[B83] Mietto B. S. de Souza B. J. Rosa P. S. Pessolani M. C. V. Lara F. A. Sarno E. N. (2020). Myelin Breakdown Favours *Mycobacterium leprae* Survival in Schwann Cells. Cell Microbiol 22 (1), e13128. 10.1111/cmi.13128 31652371

[B84] Mou Y. Dong Y. Chen Z. Denton K. R. Duff M. O. Blackstone C. (2020). Impaired Lipid Metabolism in Astrocytes Underlies Degeneration of Cortical Projection Neurons in Hereditary Spastic Paraplegia. Acta Neuropathol. Commun. 8 (1), 214. 10.1186/s40478-020-01088-0 33287888 PMC7720406

[B85] Moulton M. J. Barish S. Ralhan I. Chang J. Goodman L. D. Harland J. G. (2021). Neuronal ROS-Induced Glial Lipid Droplet Formation Is Altered by Loss of Alzheimer's Disease-Associated Genes. Proc. Natl. Acad. Sci. U S A. 118 (52), e2112095118. 10.1073/pnas.2112095118 34949639 PMC8719885

[B86] Moutinho M. Nunes M. J. Rodrigues E. (2016). Cholesterol 24-hydroxylase: Brain Cholesterol Metabolism and beyond. Biochim. Biophys. Acta 1861 (12 Pt A), 1911–1920. 10.1016/j.bbalip.2016.09.011 27663182

[B87] Muliyil S. Levet C. Düsterhöft S. Dulloo I. Cowley S. A. Freeman M. (2020). ADAM17-triggered TNF Signalling Protects the Ageing Drosophila Retina from Lipid Droplet-Mediated Degeneration. Embo j 39 (17), e104415. 10.15252/embj.2020104415 32715522 PMC7459420

[B88] Nagarajan S. R. Butler L. M. Hoy A. J. (2021). The Diversity and Breadth of Cancer Cell Fatty Acid Metabolism. Cancer Metab. 9 (1), 2. 10.1186/s40170-020-00237-2 33413672 PMC7791669

[B89] Najt C. P. Khan S. A. Heden T. D. Witthuhn B. A. Perez M. Heier J. L. (2020). Lipid Droplet-Derived Monounsaturated Fatty Acids Traffic via PLIN5 to Allosterically Activate SIRT1. Mol. Cell 77 (4), 810–824. 10.1016/j.molcel.2019.12.003 31901447 PMC7036014

[B90] Nakajima S. Gotoh M. Fukasawa K. Murakami-Murofushi K. Kunugi H. (2019). Oleic Acid Is a Potent Inducer for Lipid Droplet Accumulation through its Esterification to Glycerol by Diacylglycerol Acyltransferase in Primary Cortical Astrocytes. Brain Res. 1725, 146484. 10.1016/j.brainres.2019.146484 31562840

[B91] Narendra D. P. Guillermier C. Gyngard F. Huang X. Ward M. E. Steinhauser M. L. (2020). Coupling APEX Labeling to Imaging Mass Spectrometry of Single Organelles Reveals Heterogeneity in Lysosomal Protein Turnover. J. Cell Biol 219 (1), e201901097. 10.1083/jcb.201901097 31719114 PMC7039203

[B92] Newell C. L. Vorng J. L. MacRae J. I. Gilmore I. S. Gould A. P. (2020). Cryogenic OrbiSIMS Localizes Semi‐Volatile Molecules in Biological Tissues. Angew. Chem. Int. Ed. 59 (41), 18194–18200. 10.1002/anie.202006881 PMC7589292 32603009

[B93] Nguyen T. B. Louie S. M. Daniele J. R. Tran Q. Dillin A. Zoncu R. (2017). DGAT1-Dependent Lipid Droplet Biogenesis Protects Mitochondrial Function during Starvation-Induced Autophagy. Developmental Cell 42 (1), 9–21. e5. 10.1016/j.devcel.2017.06.003 28697336 PMC5553613

[B94] Ogrodnik M. Zhu Y. Langhi L. G. P. Tchkonia T. Krüger P. Fielder E. (2019). Obesity-Induced Cellular Senescence Drives Anxiety and Impairs Neurogenesis. Cell Metab. 29 (5), 1061–1077. e8. 10.1016/j.cmet.2018.12.008 30612898 PMC6509403

[B95] Olzmann J. A. Carvalho P. (2019). Dynamics and Functions of Lipid Droplets. Nat. Rev. Mol. Cell Biol 20 (3), 137–155. 10.1038/s41580-018-0085-z 30523332 PMC6746329

[B96] Opazo-Ríos L. Mas S. Marín-Royo G. Mezzano S. Gómez-Guerrero C. Moreno J. A. (2020). Lipotoxicity and Diabetic Nephropathy: Novel Mechanistic Insights and Therapeutic Opportunities. Ijms 21 (7), 2632. 10.3390/ijms21072632 PMC7177360 32290082

[B97] Pennetta G. Welte M. A. (2018). Emerging Links between Lipid Droplets and Motor Neuron Diseases. Developmental Cell 45 (4), 427–432. 10.1016/j.devcel.2018.05.002 29787708 PMC5988256

[B98] Petan T. (2020). Lipid Droplets in Cancer. Rev. Physiol. Biochem. Pharmacol. 10.1007/112_2020_51 33074407

[B99] Puglielli L. Konopka G. Pack-Chung E. Ingano L. A. M. Berezovska O. Hyman B. T. (2001). Acyl-coenzyme A: Cholesterol Acyltransferase Modulates the Generation of the Amyloid β-peptide. Nat. Cell Biol 3 (10), 905–912. 10.1038/ncb1001-905 11584272

[B100] Qi G. Mi Y. Shi X. Gu H. Brinton R. D. Yin F. (2021). ApoE4 Impairs Neuron-Astrocyte Coupling of Fatty Acid Metabolism. Cell Rep. 34 (1), 108572. 10.1016/j.celrep.2020.108572 33406436 PMC7837265

[B101] Raas Q. Saih F.-E. Gondcaille C. Trompier D. Hamon Y. Leoni V. (2019). A Microglial Cell Model for Acyl-CoA Oxidase 1 Deficiency. Biochim. Biophys. Acta (Bba) - Mol. Cell Biol. Lipids 1864 (4), 567–576. 10.1016/j.bbalip.2018.10.005 30312667

[B102] Rakotonirina-Ricquebourg R. Costa V. Teixeira V. (2022). Hello from the Other Side: Membrane Contact of Lipid Droplets with Other Organelles and Subsequent Functional Implications. Prog. Lipid Res. 85, 101141. 10.1016/j.plipres.2021.101141 34793861

[B103] Ralhan I. Chang C. L. Lippincott-Schwartz J. Ioannou M. S. (2021). Lipid Droplets in the Nervous System. J. Cell Biol 220 (7), e202102136. 10.1083/jcb.202102136 34152362 PMC8222944

[B104] Rambold A. S. Cohen S. Lippincott-Schwartz J. (2015). Fatty Acid Trafficking in Starved Cells: Regulation by Lipid Droplet Lipolysis, Autophagy, and Mitochondrial Fusion Dynamics. Developmental Cell 32 (6), 678–692. 10.1016/j.devcel.2015.01.029 25752962 PMC4375018

[B105] Ramosaj M. Madsen S. Maillard V. Scandella V. Sudria-Lopez D. Yuizumi N. (2021). Lipid Droplet Availability Affects Neural Stem/progenitor Cell Metabolism and Proliferation. Nat. Commun. 12 (1), 7362. 10.1038/s41467-021-27365-7 34934077 PMC8692608

[B106] Renvoisé B. Malone B. Falgairolle M. Munasinghe J. Stadler J. Sibilla C. (2016). Reep1 Null Mice Reveal a Converging Role for Hereditary Spastic Paraplegia Proteins in Lipid Droplet Regulation. Hum. Mol. Genet. 25 (23), 5111–5125. 10.1093/hmg/ddw315 27638887 PMC6078631

[B107] Roberts M. A. Olzmann J. A. (2020). Protein Quality Control and Lipid Droplet Metabolism. Annu. Rev. Cell Dev. Biol. 36, 115–139. 10.1146/annurev-cellbio-031320-101827 33021827 PMC7593838

[B108] Roy D. Tedeschi A. (2021). The Role of Lipids, Lipid Metabolism and Ectopic Lipid Accumulation in Axon Growth, Regeneration and Repair after CNS Injury and Disease. Cells 10 (5), 1078. 10.3390/cells10051078 34062747 PMC8147289

[B109] Rui Y.-N. Xu Z. Patel B. Chen Z. Chen D. Tito A. (2015). Huntingtin Functions as a Scaffold for Selective Macroautophagy. Nat. Cell Biol 17 (3), 262–275. 10.1038/ncb3101 25686248 PMC4344873

[B110] Saito K. Dubreuil V. Arai Y. Wilsch-Bräuninger M. Schwudke D. Saher G. (2009). Ablation of Cholesterol Biosynthesis in Neural Stem Cells Increases Their VEGF Expression and Angiogenesis but Causes Neuron Apoptosis. Proc. Natl. Acad. Sci. U.S.A. 106 (20), 8350–8355. 10.1073/pnas.0903541106 19416849 PMC2688855

[B111] Sanhueza M. Chai A. Smith C. McCray B. A. Simpson T. I. Taylor J. P. (2015). Network Analyses Reveal Novel Aspects of ALS Pathogenesis. Plos Genet. 11 (3), e1005107. 10.1371/journal.pgen.1005107 25826266 PMC4380362

[B112] Schuldiner M. Bohnert M. (2017). A Different Kind of Love - Lipid Droplet Contact Sites. Biochim. Biophys. Acta Mol. Cell Biol Lipids 1862 (10 Pt B), 1188–1196. 10.1016/j.bbalip.2017.06.005 28627434

[B113] Schulz J. G. Laranjeira A. Van Huffel L. Gärtner A. Vilain S. Bastianen J. (2015). Glial β-Oxidation Regulates Drosophila Energy Metabolism. Sci. Rep. 5, 7805. 10.1038/srep07805 25588812 PMC4295106

[B114] Schuurs-Hoeijmakers J. H. M. Geraghty M. T. Kamsteeg E.-J. Ben-Salem S. de Bot S. T. Nijhof B. (2012). Mutations in DDHD2, Encoding an Intracellular Phospholipase A1, Cause a Recessive Form of Complex Hereditary Spastic Paraplegia. Am. J. Hum. Genet. 91 (6), 1073–1081. 10.1016/j.ajhg.2012.10.017 23176823 PMC3516595

[B115] Scorletti E. Carr R. M. (2022). A New Perspective on NAFLD: Focusing on Lipid Droplets. J. Hepatol. 76 (4), 934–945. 10.1016/j.jhep.2021.11.009 34793866

[B116] Shibuya Y. Chang C. C. Y. Huang L.-H. Bryleva E. Y. Chang T.-Y. (2014). Inhibiting ACAT1/SOAT1 in Microglia Stimulates Autophagy-Mediated Lysosomal Proteolysis and Increases A 1-42 Clearance. J. Neurosci. 34 (43), 14484–14501. 10.1523/jneurosci.2567-14.2014 25339759 PMC4205563

[B117] Shibuya Y. Niu Z. Bryleva E. Y. Harris B. T. Murphy S. R. Kheirollah A. (2015). Acyl-coenzyme A:cholesterol Acyltransferase 1 Blockage Enhances Autophagy in the Neurons of Triple Transgenic Alzheimer's Disease Mouse and Reduces Human P301L-Tau Content at the Presymptomatic Stage. Neurobiol. Aging 36 (7), 2248–2259. 10.1016/j.neurobiolaging.2015.04.002 25930235 PMC4457653

[B118] Shimabukuro M. K. Langhi L. G. P. Cordeiro I. Brito J. M. Batista C. M. d. C. Mattson M. P. (2016). Lipid-laden Cells Differentially Distributed in the Aging Brain Are Functionally Active and Correspond to Distinct Phenotypes. Sci. Rep. 6, 23795. 10.1038/srep23795 27029648 PMC4814830

[B119] Singh R. Kaushik S. Wang Y. Xiang Y. Novak I. Komatsu M. (2009). Autophagy Regulates Lipid Metabolism. Nature 458 (7242), 1131–1135. 10.1038/nature07976 19339967 PMC2676208

[B120] Smolič T. Tavčar P. Horvat A. Černe U. Halužan Vasle A. Tratnjek L. (2021). Astrocytes in Stress Accumulate Lipid Droplets. Glia 69 (6), 1540–1562. 10.1002/glia.23978 33609060 PMC8248329

[B121] Soste M. Charmpi K. Lampert F. Gerez J. A. van Oostrum M. Malinovska L. (2019). Proteomics-Based Monitoring of Pathway Activity Reveals that Blocking Diacylglycerol Biosynthesis Rescues from Alpha-Synuclein Toxicity. Cell Syst. 9 (3), 309–320. 10.1016/j.cels.2019.07.010 31521608 PMC6859835

[B122] Staurenghi E. Giannelli S. Testa G. Sottero B. Leonarduzzi G. Gamba P. (2021). Cholesterol Dysmetabolism in Alzheimer's Disease: A Starring Role for Astrocytes? Antioxidants (Basel) 10 (12). 10.3390/antiox10121890 PMC8750262 34943002

[B123] Steinhauser M. L. Bailey A. P. Senyo S. E. Guillermier C. Perlstein T. S. Gould A. P. (2012). Multi-isotope Imaging Mass Spectrometry Quantifies Stem Cell Division and Metabolism. Nature 481 (7382), 516–519. 10.1038/nature10734 22246326 PMC3267887

[B124] Stok R. Ashkenazi A. (2020). Lipids as the Key to Understanding α-synuclein Behaviour in Parkinson Disease. Nat. Rev. Mol. Cell Biol 21 (7), 357–358. 10.1038/s41580-020-0235-y 32203270

[B125] Stoll E. A. Makin R. Sweet I. R. Trevelyan A. J. Miwa S. Horner P. J. (2015). Neural Stem Cells in the Adult Subventricular Zone Oxidize Fatty Acids to Produce Energy and Support Neurogenic Activity. Stem Cells 33 (7), 2306–2319. 10.1002/stem.2042 25919237 PMC4478223

[B126] Suzuki M. Fujikake N. Takeuchi T. Kohyama-Koganeya A. Nakajima K. Hirabayashi Y. (2015). Glucocerebrosidase Deficiency Accelerates the Accumulation of Proteinase K-Resistant α-synuclein and Aggravates Neurodegeneration in aDrosophilamodel of Parkinson's Disease. Hum. Mol. Genet. 24 (23), 6675–6686. 10.1093/hmg/ddv372 26362253

[B127] Tadepalle N. Rugarli E. I. (2021). Lipid Droplets in the Pathogenesis of Hereditary Spastic Paraplegia. Front. Mol. Biosci. 8, 673977. 10.3389/fmolb.2021.673977 34041268 PMC8141572

[B128] Taïb B. Aboussalah A. M. Moniruzzaman M. Chen S. Haughey N. J. Kim S. F. (2019). Lipid Accumulation and Oxidation in Glioblastoma Multiforme. Sci. Rep. 9 (1), 19593. 10.1038/s41598-019-55985-z 31863022 PMC6925201

[B129] Tamosaityte S. Galli R. Uckermann O. Sitoci-Ficici K. H. Koch M. Later R. (2016). Inflammation-related Alterations of Lipids after Spinal Cord Injury Revealed by Raman Spectroscopy. J. Biomed. Opt. 21 (6), 61008. 10.1117/1.JBO.21.6.061008 27146789

[B130] Taylor M. J. Lukowski J. K. Anderton C. R. (2021). Spatially Resolved Mass Spectrometry at the Single Cell: Recent Innovations in Proteomics and Metabolomics. J. Am. Soc. Mass. Spectrom. 32 (4), 872–894. 10.1021/jasms.0c00439 33656885 PMC8033567

[B131] Teixeira V. Maciel P. Costa V. (2021). Leading the Way in the Nervous System: Lipid Droplets as New Players in Health and Disease. Biochim. Biophys. Acta (Bba) - Mol. Cell Biol. Lipids 1866 (1), 158820. 10.1016/j.bbalip.2020.158820 33010453

[B132] Thiam A. R. Ikonen E. (2021). Lipid Droplet Nucleation. Trends Cell Biol. 31 (2), 108–118. 10.1016/j.tcb.2020.11.006 33293168

[B133] Van Den Brink D. M. Cubizolle A. Chatelain G. Davoust N. Girard V. Johansen S. (2018). Physiological and Pathological Roles of FATP-Mediated Lipid Droplets in Drosophila and Mice Retina. Plos Genet. 14 (9), e1007627. 10.1371/journal.pgen.1007627 30199545 PMC6147681

[B134] van der Kant R. Langness V. F. Herrera C. M. Williams D. A. Fong L. K. Leestemaker Y. (2019). Cholesterol Metabolism Is a Druggable Axis that Independently Regulates Tau and Amyloid-β in iPSC-Derived Alzheimer's Disease Neurons. Cell Stem Cell 24 (3), 363–375. 10.1016/j.stem.2018.12.013 30686764 PMC6414424

[B135] Vincent B. M. Tardiff D. F. Piotrowski J. S. Aron R. Lucas M. C. Chung C. Y. (2018). Inhibiting Stearoyl-CoA Desaturase Ameliorates α-Synuclein Cytotoxicity. Cell Rep. 25 (10), 2742–2754. 10.1016/j.celrep.2018.11.028 30517862

[B136] Walther T. C. Chung J. Farese R. V. Jr. (2017). Lipid Droplet Biogenesis. Annu. Rev. Cell Dev. Biol. 33, 491–510. 10.1146/annurev-cellbio-100616-060608 28793795 PMC6986389

[B137] Walther T. C. Farese R. V. Jr. (2012). Lipid Droplets and Cellular Lipid Metabolism. Annu. Rev. Biochem. 81, 687–714. 10.1146/annurev-biochem-061009-102430 22524315 PMC3767414

[B138] Wang L. Xing X. Zeng X. Jackson S. R. TeSlaa T. Al-Dalahmah O. (2022). Spatially Resolved Isotope Tracing Reveals Tissue Metabolic Activity. Nat. Methods 19 (2), 223–230. 10.1038/s41592-021-01378-y 35132243 PMC10926149

[B139] Wat L. W. Chao C. Bartlett R. Buchanan J. L. Millington J. W. Chih H. J. (2020). A Role for Triglyceride Lipase Brummer in the Regulation of Sex Differences in Drosophila Fat Storage and Breakdown. Plos Biol. 18 (1), e3000595. 10.1371/journal.pbio.3000595 31961851 PMC6994176

[B140] Welte M. A. Gould A. P. (2017). Lipid Droplet Functions beyond Energy Storage. Biochim. Biophys. Acta Mol. Cell Biol Lipids 1862 (10 Pt B), 1260–1272. 10.1016/j.bbalip.2017.07.006 28735096 PMC5595650

[B141] Wilfling F. Haas J. T. Walther T. C. Jr R. V. F. Jr (2014). Lipid Droplet Biogenesis. Curr. Opin. Cell Biol. 29, 39–45. 10.1016/j.ceb.2014.03.008 24736091 PMC4526149

[B142] Windpassinger C. Auer-Grumbach M. Irobi J. Patel H. Petek E. Hörl G. (2004). Heterozygous Missense Mutations in BSCL2 Are Associated with Distal Hereditary Motor Neuropathy and Silver Syndrome. Nat. Genet. 36 (3), 271–276. 10.1038/ng1313 14981520

[B143] Wu X. Geng F. Cheng X. Guo Q. Zhong Y. Cloughesy T. F. (2020). Lipid Droplets Maintain Energy Homeostasis and Glioblastoma Growth via Autophagic Release of Stored Fatty Acids. iScience 23 (10), 101569. 10.1016/j.isci.2020.101569 33083736 PMC7549116

[B144] Xie Z. Jones A. Deeney J. T. Hur S. K. Bankaitis V. A. (2016). Inborn Errors of Long-Chain Fatty Acid β-Oxidation Link Neural Stem Cell Self-Renewal to Autism. Cell Rep. 14 (5), 991–999. 10.1016/j.celrep.2016.01.004 26832401 PMC4749429

[B145] Xu Y. Propson N. E. Du S. Xiong W. Zheng H. (2021). Autophagy Deficiency Modulates Microglial Lipid Homeostasis and Aggravates Tau Pathology and Spreading. Proc. Natl. Acad. Sci. U S A. 118 (27), e2023418118. 10.1073/pnas.2023418118 34187889 PMC8271658

[B146] Yang C. Wang X. Wang J. Wang X. Chen W. Lu N. (2020). Rewiring Neuronal Glycerolipid Metabolism Determines the Extent of Axon Regeneration. Neuron 105 (2), 276–292. e5. 10.1016/j.neuron.2019.10.009 31786011 PMC6975164

[B147] Yang D. S. Stavrides P. Saito M. Kumar A. Rodriguez-Navarro J. A. Pawlik M. (2014). Defective Macroautophagic Turnover of Brain Lipids in the TgCRND8 Alzheimer Mouse Model: Prevention by Correcting Lysosomal Proteolytic Deficits. Brain 137 (Pt 12), 3300–3318. 10.1093/brain/awu278 25270989 PMC4240291

[B148] Yang L. Liang J. Lam S. M. Yavuz A. Shui G. Ding M. (2020). Neuronal Lipolysis Participates in PUFA-Mediated Neural Function and Neurodegeneration. EMBO Rep. 21 (11), e50214. 10.15252/embr.202050214 33034119 PMC7645260

[B149] Yeshaw W. M. van der Zwaag M. Pinto F. Lahaye L. L. Faber A. I. Gómez-Sánchez R. (2019). Human VPS13A Is Associated with Multiple Organelles and Influences Mitochondrial Morphology and Lipid Droplet Motility. Elife 8, e43561. 10.7554/eLife.43561 30741634 PMC6389287

[B150] Yoon H. Shaw J. L. Haigis M. C. Greka A. (2021). Lipid Metabolism in Sickness and in Health: Emerging Regulators of Lipotoxicity. Mol. Cell 81 (18), 3708–3730. 10.1016/j.molcel.2021.08.027 34547235 PMC8620413

[B151] Zhuang H. Yao X. Li H. Li Q. Yang C. Wang C. (2022). Long-term High-Fat Diet Consumption by Mice throughout Adulthood Induces Neurobehavioral Alterations and Hippocampal Neuronal Remodeling Accompanied by Augmented Microglial Lipid Accumulation. Brain Behav. Immun. 100, 155–171. 10.1016/j.bbi.2021.11.018 34848340

[B152] Züchner S. Wang G. Tran-Viet K.-N. Nance M. A. Gaskell P. C. Vance J. M. (2006). Mutations in the Novel Mitochondrial Protein REEP1 Cause Hereditary Spastic Paraplegia Type 31. Am. J. Hum. Genet. 79 (2), 365–369. 10.1086/505361 16826527 PMC1559498

